# Editorial: Perspectives for natural language processing between AI, linguistics and cognitive science

**DOI:** 10.3389/frai.2022.1059998

**Published:** 2022-11-03

**Authors:** Alessandro Lenci, Sebastian Padó

**Affiliations:** ^1^Computational Linguistics Laboratory, University of Pisa, Pisa, Italy; ^2^Institut für Maschinelle Sprachverarbeitung, University of Stuttgart, Stuttgart, Germany

**Keywords:** artificial intelligence, natural language processing, linguistics, interdisciplinary, cognitive science

Natural Language Processing (NLP) today—like most of Artificial Intelligence (AI)—is much more of an “engineering” discipline than it originally was, when it sought to develop a general theory of human language understanding that not only translates into language technology, but that is also linguistically meaningful and cognitively plausible.

At first glance, this trend seems to be connected to the rapid development in the last 10 years that was driven to a large extent by the adoption of deep learning techniques. However, it can be argued that the move toward deep learning has the potential of bringing NLP back to its roots after all. Some recent activities and findings in this direction include: Techniques like multi-task learning have been used to integrate cognitive data as supervision in NLP tasks (Barrett et al., 2016); Pre-training/fine-tuning regimens are potentially interpretable in terms of cognitive mechanisms like general competencies applied to specific tasks (Flesch et al., 2018); The ability of modern models for ‘few-shot' or even ‘zero-shot' performance on novel tasks mirrors human performance (Srivastava et al., 2018); Evidence of unsupervised structure learning in current neural network architectures that mirrors classical linguistic structures (Hewitt and Manning, 2019; Tenney et al., 2019).

In terms of developing systems endowed with natural language capabilities, the last generation of neural network architectures has allowed AI and NLP to make unprecedented progress. Such systems (e.g., the GPT family) are typically trained with huge computational infrastructures on large amounts of textual data from which they acquire knowledge thanks to their extraordinary ability to record and generalize the statistical patterns found in data. However, the debate about the human-like semantic abilities that such “juggernaut models” really acquire is still wide open. In fact, despite the figures typically reported to show the success of AI on various benchmarks, other research argues that their semantic competence is still very brittle (Lake and Baroni, 2018; Bender and Koller, 2020; Ravichander et al., 2020). Thus, an important limitation of current AI research is the lack of attention to the mechanisms behind human language understanding. The latter does not only consist of a brute-force, data-intensive processing of statistical regularities but it is also governed by complex inferential mechanisms that integrate linguistic information and contextual knowledge coming from different sources and potentially different modalities.

The current Research Topic was conceived on the assumption that the possibility for new breakthroughs in the study of human and machine intelligence calls for a new alliance between NLP, AI, and linguistic and cognitive research. The current computational paradigms can offer new ways to explore human language learning and processing, while linguistic and cognitive research can highlight those aspects of human intelligence that systems need to model or incorporate within their architectures.

We are very happy to present seven articles that embody this promise in different ways.

Two papers focus on the use of large neural language models to model aspects of natural language syntax, arguably a cornerstone of human linguistic competence, and therefore a target of much research in recent years. Oh et al.'s *Comparison of structural parsers and neural language models as surprisal estimators* contrasts the current standard architecture—neural parsers trained in a purely data-driven fashion—against a parser incorporating linguistic generalizations and find a better fit with various reading time measures for the latter. Kulmizev and Nivre's *Schrödinger's tree–on syntax and neural language models* makes a methodological contribution, sounding a note of caution about the current state of affairs. They point out the large impact that choices regarding experimental design and evaluation measures have on the study of syntactic generalizations in neural parsers.

Three more papers are concerned primarily with natural language semantics, a long-standing multi-dimensional problem that has so far resisted comprehensive modeling. The papers bring different methods to bear on this topic: Brown et al.'s *Semantic representations for NLP using VerbNet and the generative lexicon* continues a long tradition of careful linguistic modeling work, demonstrating how the combination of semantic theories and carefully curated lexical resources can provide computational predictions of event semantics with broad coverage. In contrast, Schulte im Walde and Frassinelli's *Distributional measures of semantic abstraction* proposes a decomposition of the concept of semantic abstraction into the two dimensions of abstractness/concreteness and specificity/generality and demonstrates that distributional corpus evidence can model both sub-aspects convincingly. The third paper, Stevenson and Merlo's *Beyond the benchmarks: toward human-like lexical representations*, is again located at the methodological level, offering a critical review of current computational investigations into lexical representation and perspectives looking forward. In particular, they stress the need for models able to address the rich structure of lexical meanings, which is still only partially tackled by mainstream computational semantic approaches, including those based on word embeddings.

The two final papers take seriously the idea of multimodality, extending their reach beyond textual data, as a strategy to address long-standing challenges in natural language processing. Bruera and Poesio's *Exploring the representations of individual entities in the brain combining eeg and distributional semantics* compare corpus-based and EEG-based embeddings for entities, paving the way toward a better understanding of the relationship between online and offline representations. Finally, Krishnaswamy and Pustejovsky's *Affordance embeddings for situated language understanding*” argues that grounding of language in concrete situations, whether real or simulated, is a crucial step toward generalized learning, and demonstrate this claim with a model capable of learning properties of novel objects.

Taken together, we believe that these papers offer important contributions to the state of the art and open promising directions for future research. Despite their different approaches and perspectives, all papers support the same conclusion: It is time for a new alliance between AI, linguistics, and cognitive science, because only from their synergistic efforts and mutual feeding can we hope to achieve significant breakthroughs in the computational modeling of human intelligence and of natural language in particular. In closing, we would like to express our gratitude to the reviewers for their timely and insightful comments, and to the authors that have engaged with them a constructive scientific discussion.

## Author contributions

AL and SP wrote the editorial together. Both authors contributed to the article and approved the submitted version.

## Conflict of interest

The authors declare that the research was conducted in the absence of any commercial or financial relationships that could be construed as a potential conflict of interest.

## Publisher's note

All claims expressed in this article are solely those of the authors and do not necessarily represent those of their affiliated organizations, or those of the publisher, the editors and the reviewers. Any product that may be evaluated in this article, or claim that may be made by its manufacturer, is not guaranteed or endorsed by the publisher.
